# Antimicrobial Susceptibility of Invasive *Streptococcus pyogenes* Isolates in Germany during 2003-2013

**DOI:** 10.1371/journal.pone.0137313

**Published:** 2015-09-04

**Authors:** Matthias Imöhl, Mark van der Linden

**Affiliations:** Institute of Medical Microbiology and National Reference Center for Streptococci, University Hospital RWTH Aachen, Aachen, Germany; Faculdade de Medicina de Lisboa, PORTUGAL

## Abstract

A nationwide laboratory-based surveillance study of invasive *S*. *pyogenes* infections was conducted in Germany. Invasive isolates (*n* = 1,281) were obtained between 2003 and 2013. All isolates were susceptible to penicillin, cefotaxime and vancomycin. Tetracycline showed the highest rate of resistant or intermediate resistant isolates with 9.8%, followed by macrolides (4.0%), trimethoprim/sulfamethoxazole (SXT) (1.9%), levofloxacin (1.3%), chloramphenicol (0.9%) and clindamycin (0.7%). The most prominent trends were the appearance of levofloxacin non-susceptible isolates since 2011, and an increase of SXT non-susceptibility since 2012.

## Introduction

*Streptococcus pyogenes* (Lancefield group A streptococcus; GAS) is a major human pathogen and responsible for a wide range of both suppurative and non-suppurative diseases, e.g. pharyngitis, erysipelas, septicaemia, meningitis, pneumonia and the notably severe manifestations necrotising fasciitis (NF) and streptococcal toxic shock syndrome (STSS). Suppurative infections and also post-infection sequelae, e.g. acute rheumatic fever, rheumatic heart disease and glomerulonephritis, result in substantial human morbidity [1]. Invasive infections caused by *S*. *pyogenes* (iGAS) have been increasingly reported since the mid- to late 1980s [2]. Recent upsurges in iGAS infections were reported from Sweden [3], Ireland [4, 5] and England [6]. The global burden of invasive *S*. *pyogenes* disease is estimated to be high, with at least 663,000 new cases and 163,000 deaths worldwide each year [7].

The resistance rates of *S*. *pyogenes* to several antibiotics vary considerably worldwide. Resistance rates from 2% to 98% have been reported for macrolides. While in several European countries, an increase of macrolide resistance has been described during the last 10–20 years, recently a decrease has been noted in some of these countries [8]. However, *S*. *pyogenes* still remains uniformly susceptible to penicillin, which is the antimicrobial of choice for the treatment of GAS infections. In case of penicillin allergy, a first-generation cephalosporin (for patients not anaphylactically sensitive), macrolides (clarithromycin or azithromycin) or lincosamides (clindamycin) are the recommended primary alternatives [8]. In patients with severe penicillin hypersensitivity, vancomycin, linezolid and quinupristin/dalfopristin have been described as further alternatives. Clindamycin combined with penicillin is the first choice for the treatment of life-threatening GAS infections, such as necrotizing fasciitis, STSS, meningitis, pneumonia. Clindamycin has been shown to be an inhibitor of the production of streptococcal superantigens and other virulence factors, such as the M protein, and to improve the efficacy of the penicillin/clindamycin combination compared with the β-lactam alone. Linezolid possibly shares the beneficial effect of clindamycin as a protein inhibitor, although there are currently only few data to support this [8, 9]. Further alternative or supplemental antibiotics that have a clinical indication for GAS infections reported in this study include cefotaxime, levofloxacin, chloramphenicol, tetracycline and trimethoprim/sulfamethoxazole (SXT).

The two main mechanisms of macrolide resistance in GAS isolates are a target site modification, which prevents the binding of the antimicrobial to the ribosome, and an active efflux of the antimicrobial, which reduces its concentration in the cytoplasm.

The target site modification is due to a 23S rRNA methylase that mediates ribosomal modification of the macrolide-binding site [10]. This results in cross-resistance to all macrolides, lincosamides and streptogramins B. This is called the MLS (or MLSB) phenotype which can be expressed either constitutively (cMLS) or in an inducible manner (iMLS) [8, 11, 12], and is encoded by the *erm* genes (erythromycin ribosome methylase) [8].

The efflux mechanism consists of a membrane-spanning pump, which reduces the intracellular antibiotic concentration to subtoxic levels [8]. In streptococci, the efflux mechanism confers low to moderate levels of resistance to 14- and 15-membered lactone ring macrolides, but not to 16-member macrolides nor to lincosamides or streptogramins B. This is called the M phenotype [8]. In GAS isolates, these pumps are generally encoded by the *mef*(A) gene, although other *mef* variants have been recognized in some strains [8].

The present investigation was conducted to simplify the choice of antibiotics, especially in cases where penicillin is not an option for therapy. The study rests upon data from invasive *S*. *pyogenes* strains collected in nationwide, voluntary, laboratory-based surveillance in Germany from 2003 to 2013.

## Materials and Methods

### Study design

German microbiological laboratories were invited to send their isolates to the German National Reference Center for Streptococci (NRCS; Aachen, Germany). Isolates were included into the study when they met the criteria of an invasive infection according to the definition of the Working Group on Severe Streptococcal Infections 1993 [13]. The present study is part of an ongoing surveillance and data on iGAS resistance from 2003 to 2007 have been previously published by our group [14]. However, in the previous publication, no detailed data on variation in resistance from year to year were given.

### Microbiological investigations

Isolates were identified by *β*-haemolysis on sheep blood agar, Lancefield antigen grouping using a commercially available agglutination technique (Slidex Streptokit, bioMérieux, Marcy-L’Etoile, France; Prolex Streptococcal Grouping Latex Kits, Pro-Lab Diagnostics, Richmond Hill, Canada), the pyrrolidonyl-arylamidase (PYR) test, and the detection of *emm* genes by PCR using ‘all M primers’ as described previously [15]. Antibiotic susceptibility testing was performed using the micro-broth dilution method and susceptibility categorization as recommended by the Clinical and Laboratory Standards Institute (CLSI) [16]. Since the MIC testing strictly referred to the CLSI recommendations but no MIC interpretive criteria for SXT were specified by the CLSI [16], the EUCAST breakpoints were used to estimate the resistance rate for SXT for reasons of exploratory analysis only [17]. Macrolide resistance was investigated using either erythromycin or clarithromycin. Clarithromycin was most frequently used from 2004–2011, whereas erythromycin was used before 2004 and after 2011. Macrolide non-susceptible isolates underwent further examination and were phenotyped using a modification of the erythromycin-clindamycin double-disk test as described by Seppälä et al [18] or the triple-disk test (erythromycin and clindamycin plus josamycin) as described by Giovanetti et al [19] and classified as M phenotype or inducibly (iMLS) or constitutively (cMLS) coresistant to macrolide, lincosamide and streptogramin B antibiotics. Furthermore the isolates were genotyped by PCR as described previously by our group [12]. The interpretation of clindamycin resistance is based on the results of the MIC testing only; however, all macrolide non-susceptible isolates were phenotyped / genotyped as described above.

### Statistical Analysis

Statistical testing was performed using R software(version 3.1.1, 2014). Fisher’s Exact Test was used to measure differences in proportions, and results were considered significant at p≤0.05.

### Ethical Statement

An ethical approval or patients’ consent was not required since the study only includes microbiological samples sent to the German National Reference Center for Streptococci on an anonymized basis by the sending microbiological laboratories, and did not involve human subjects or material.

## Results

A total of 1,281 iGAS samples were collected between 1 January 2003 and 31 December 2013. The numbers of included cases for each year varied between 74 and 169 cases (median: 116 cases).

All isolates were susceptible to penicillin, cefotaxime and vancomycin. Six of the antibiotics tested (chloramphenicol, clindamycin, levofloxacin, macrolides, tetracycline and SXT) were observed to have some level of resistance (Table 1). Tetracycline showed the highest rate of resistant or intermediate isolates with 9.8% on average from 2003 to 2013, followed by macrolides (4.0%), SXT (1.9%), levofloxacin (1.3%), chloramphenicol (0.9%) and clindamycin (0.7%). The most prominent trends were the appearance of levofloxacin non-susceptible isolates in 2011, and the increase of SXT non-susceptibility in 2012. In 2011 and 2013, levofloxacin non-susceptible isolates were found significantly more often than in all other study years (*p* = 6.15x10^-5^ and *p* = 1.82x10^-2^, respectively). In 2012 and 2013, SXT non-susceptibility also reached statistical significance (*p* = 9.16x10^-8^ and *p* = 3.12x10^-2^, respectively). 2013 also saw statistically significant increases in clindamycin-resistant and chloramphenicol-resistant isolates (*p* = 3.21x10^-3^ and *p* = 9.49x10^-3^, respectively).

**Table 1:** Susceptibility of iGAS isolates to various antibiotics in Germany from 2003 to 2013. All isolates were susceptible to penicillin, cefotaxime and vancomycin.

	**Macrolides** ^1^							**Clindamycin**							**Levofloxacin**						
**Year**	**S (n)**	**S (%)**	**I (n)**	**I (%)**	**R (n)**	**R (%)**	**n**	**S (n)**	**S (%)**	**I (n)**	**I (%)**	**R (n)**	**R (%)**	**n**	**S (n)**	**S (%)**	**I (n)**	**I (%)**	**R (n)**	**R (%)**	**n**
2003	67	94.4	0	0.0	4	5.6	71	71	100.0	0	0.0	0	0.0	71	69	100.0	0	0.0	0	0.0	69
2004	154	96.9	0	0.0	5	3.1	159	157	98.7	0	0.0	2	1.3	159	159	100.0	0	0.0	0	0.0	159
2005	80	89.9	2	2.2	7	7.9	89	89	100.0	0	0.0	0	0.0	89	89	100.0	0	0.0	0	0.0	89
2006	84	95.5	0	0.0	4	4.5	88	88	100.0	0	0.0	0	0.0	88	88	100.0	0	0.0	0	0.0	88
2007	81	95.3	0	0.0	4	4.7	85	86	100.0	0	0.0	0	0.0	86	86	100.0	0	0.0	0	0.0	86
2008	104	97.2	0	0.0	3	2.8	107	107	100.0	0	0.0	0	0.0	107	107	100.0	0	0.0	0	0.0	107
2009	112	96.6	0	0.0	4	3.4	116	116	100.0	0	0.0	0	0.0	116	116	100.0	0	0.0	0	0.0	116
2010	127	97.7	0	0.0	3	2.3	130	129	99.2	0	0.0	1	0.8	130	130	100.0	0	0.0	0	0.0	130
2011	115	95.8	0	0.0	5	4.2	120	120	100.0	0	0.0	0	0.0	120	112	93.3	7	5.8	1	0.8	120
2012	129	98.5	0	0.0	2	1.5	131	130	99.2	0	0.0	1	0.8	131	128	97.7	3	2.3	0	0.0	131
2013	161	95.3	0	0.0	8	4.7	169	164	97.0	0	0.0	5	3.0	169	163	96.4	5	3.0	1	0.6	169
**Total**	**1214**	**96.0**	**2**	**0.2**	**49**	**3.9**	**1265**	**1257**	**99.3**	**0**	**0.0**	**9**	**0.7**	**1266**	**1247**	**98.7**	**15**	**1.2**	**2**	**0.2**	**1264**
	**Chloramphenicol**							**Tetracycline**							**SXT** ^2^						
**Year**	**S (n)**	**S (%)**	**I (n)**	**I (%)**	**R (n)**	**R (%)**	**n**	**S (n)**	**S (%)**	**I (n)**	**I (%)**	**R (n)**	**R (%)**	**n**	**S (n)**	**S (%)**	**I (n)**	**I (%)**	**R (n)**	**R (%)**	**n**
2003	68	98.6	0	0.0	1	1.4	69	60	84.5	0	0.0	11	15.5	71	69	100.0	0	0.0	0	0.0	69
2004	159	100.0	0	0.0	0	0.0	159	147	92.5	0	0.0	12	7.5	159	158	100.0	0	0.0	0	0.0	158
2005	88	98.9	0	0.0	1	1.1	89	77	86.5	0	0.0	12	13.5	89	89	100.0	0	0.0	0	0.0	89
2006	88	100.0	0	0.0	0	0.0	88	80	90.9	0	0.0	8	9.1	88	87	98,9	0	0.0	1	1.1	88
2007	86	100.0	0	0.0	0	0.0	86	78	91.8	0	0.0	7	8.2	85	83	100.0	0	0.0	0	0.0	83
2008	106	100.0	0	0.0	0	0.0	106	97	90.7	0	0.0	10	9.3	107	103	100.0	0	0.0	0	0.0	103
2009	116	100.0	0	0.0	0	0.0	116	101	87.1	0	0.0	15	12.9	116	114	98,3	1	0.9	1	0.9	116
2010	129	99.2	0	0.0	1	0.8	130	122	93.8	1	0.8	7	5.4	130	130	100.0	0	0.0	0	0.0	130
2011	120	100.0	0	0.0	0	0.0	120	108	90.0	0	0.0	12	10.0	120	119	99.2	1	0.8	0	0.0	120
2012	128	97.7	2	1.5	1	0.8	131	120	91.6	1	0.8	10	7.6	131	118	90.1	8	6.1	5	3.8	131
2013	164	97.0	3	1.8	2	1.2	169	150	88.8	0	0.0	19	11.2	169	160	95.8	3	1.8	4	2.4	167
**Total**	**1252**	**99.1**	**5**	**0.4**	**6**	**0.5**	**1263**	**1140**	**90.2**	**2**	**0.2**	**123**	**9.7**	**1265**	**1230**	**98.1**	**13**	**1.0**	**11**	**0.9**	**1254**

^1^Macrolides: Erythromycin or Clarithromycin;

^2^SXT susceptibility according to the EUCAST breakpoints. Since the MIC testing of the isolates strictly referred to the CLSI recommendations, the EUCAST breakpoints were used for reasons of comparison only.

For some isolates (n = 35) susceptibility testing was not performed for all nine antibiotics.

All macrolide-susceptible isolates were also susceptible to clindamycin (*n* = 1,214). Among the 51 macrolide non-susceptible isolates, 42 were susceptible and 9 were resistant to clindamycin. The macrolide resistance phenotypes and corresponding genotypes of macrolide non-susceptible iGAS isolates in Germany from 2003 to 2013 are shown in Table 2. The most frequent macrolide resistance phenotype was the M-phenotype (*n* = 26), followed in frequency by iMLS (*n* = 16) and cMLS (*n* = 9). The incidence of *mef*(A) (*n* = 14), *mef*(E) (*n* = 12), *mef*(E) and *erm*(B) (*n* = 13) and *erm*(B) (*n* = 12) was approximately similar. The most common co-resistances observed were tetracycline and macrolides (*n* = 15), tetracycline and macrolides and clindamycin (*n* = 4) and tetracycline and macrolides and chloramphenicol (*n* = 4).

**Table 2:** Macrolide resistance phenotypes and corresponding genotypes of macrolide non-susceptible iGAS isolates in Germany in the years 2003–2013.

Macrolide resistance phenotype	Macrolide resistance genotype (*n*)				Total
	*mef*(A)	*mef*(E)	*mef*(E) *+ erm*(B)	*erm*(B)	
**M**	14	12	0	0	26
**iMLS**	0	0	10	6	16
**cMLS**	0	0	3	6	9
**Total**	14	12	13	12	51

## Discussion

In this paper we present the results of 11 years of surveillance of iGAS disease in Germany. In contrast to other streptococci, *S*. *pyogenes* has to date remained universally susceptible to penicillin. All isolates tested in this study were susceptible to penicillin, cefotaxime and vancomycin.

In the present study, 4.0% of our iGAS isolates were macrolide resistant or intermediate, which is comparable to values reported from Finland (1.5%) [20] and Norway (3.4%) [21], and comparably low in a broader international context [8]. High rates of macrolide resistance in Europe have been found in Spain (17% [22]), Italy (26.5% in 1994–1996 to 18.9% in 2003–2005 [23]) and Poland (9.8% [24]). However, recently a decrease in macrolide resistance has been noted in some countries, mostly in Europe [8]. A detailed review on the prevalence of macrolide-resistant *S*. *pyogenes* isolates and the underlying dominant macrolide resistance phenotypes in multicenter studies worldwide has been published recently by Silva-Costa et al [8]. Studies from multiple countries report significant temporal changes in the prevalence of macrolide resistance phenotypes [8]. Clindamycin resistance (0.7%) is within the range reported in Finland, Germany and Norway (0.5%-2.3%) [14, 20, 21], whereas slightly higher rates of resistance have been found in Poland (4.9%) [24] and France (5.4%) [25]. The interpretation of clindamycin resistance in our study as listed in Table 1 is based on the results of the MIC testing only, i.e. only cMLS isolates were counted as resistant. However, since among the 4.0% of the macrolide non-susceptible iGAS isolates, around one third (31.4%) showed an inducible clindamycin resistance (iMLS), the potential clindamycin resistance is around 1.3% higher. For treatment purposes, an interpretative assessment of clindamycin resistance is recommended, at which iMLS isolates should be reported as resistant to clindamycin with a comment that these isolates are presumed to be resistant based on inducible clindamycin resistance.

Tetracycline shows the highest rate of non-susceptible isolates in this study among the antibiotics tested (9.8% on average from 2003 to 2013). This rate is within (6.1%, Norway [21]; 8%, Denmark [26]; 10.5%, Portugal [27]; 11.6%, Germany [14]; 13%, Spain [22]), below (16%, Finland [20]; 27.2%, Israel [28]) or far below (46.3%, Poland [24]) the range reported from other countries.

Levofloxacin non-susceptible isolates were found only during the last three years of the study, resulting in an average non-susceptibility rate of 1.3%. Reports on levofloxacin non-susceptibility data, especially among iGAS isolates, are rare. In Portugal, reduced susceptibility to levofloxacin was reported for the first time during 2006–2009, resulting in a non-susceptibility rate of 2% among invasive isolates [27].

*Streptococcus pyogenes* is commonly believed to be resistant to SXT, resulting in skepticism about using SXT for skin and soft tissue infections where *S*. *pyogenes* is involved. The infrequent reports of susceptibility of *S*. *pyogenes* to SXT demonstrate resistance rates ranging from 0% to 100% depending on growth medium and testing conditions used [29]. Most likely these variations in results are due to the methodology of testing, especially since all of the studies reporting high resistance rates either used media known to have high concentrations of thymidine or did not provide details of the medium used. Thymidine allows *S*. *pyogenes* to bypass the sulfur-mediated inhibition of folate metabolism and, historically, has resulted in apparently reduced susceptibility of *S*. *pyogenes* to sulphur antibiotics. As standardization to ensure a low thymidine concentration in Mueller-Hinton medium was introduced first in 2006, it is likely that studies prior to this may not have controlled for thymidine content [29]. Since the CLSI specifies no MIC interpretive criteria for SXT, we used the EUCAST breakpoints to estimate the resistance rate for reasons of exploratory analysis in our study. The non-susceptibility rate in our study is lower than in other studies published since 2006 (India 6.7% and 21.8%, Nepal 71%), as summarized by Bowen et al [29]. However, we observed a considerable increase in non-susceptibility in the last two study years that should be monitored in the future. This is especially important in the era of rising MRSA prevalence. *S*. *pyogenes* and *S*. *aureus* are frequently copathogens in skin and soft tissue infections. More clinical trials for the treatment of these infections with SXT are desirable [29].

Our current study was conducted to provide information on iGAS antimicrobial resistance in Germany and help in clinical decision-making to initiate an effective antibiotic treatment, especially in cases of iGAS infections where standard therapy regimens may not be an option. Fortunately, the overall responsiveness to antibiotics still is favorable for *S*. *pyogenes* in Germany, and the low non-susceptibility rates observed in our study support the administration of penicillin combined with clindamycin (non-susceptibility 0.7%) as first-line antimicrobial agents in life-threatening GAS infections, as recommended in current guidelines [9].

However, the importance of invasive *S*. *pyogenes* disease and the differing developments of iGAS antimicrobial resistance throughout the world require further surveillance.
